# Pre-zygotic isolation in the macroalgal genus *Fucus* from four contact zones spanning 100–10 000 years: a tale of reinforcement?

**DOI:** 10.1098/rsos.140538

**Published:** 2015-02-11

**Authors:** G. Hoarau, J. A. Coyer, M. C. W. G. Giesbers, A. Jueterbock, J. L. Olsen

**Affiliations:** 1 Faculty of Aquaculture and Biosciences, University of Nordland, Bodø 8049, Norway; 2 Shoals Marine Laboratory, Cornell University, 400 Little Harbor Road, Portsmouth, NH, USA; 3 Marine Benthic Ecology and Evolution Group, Centre for Ecological and Evolutionary Studies, University of Groningen, Nijenborgh 7, Groningen 9747 AG, The Netherlands

**Keywords:** introgression, hybridization, hybrid zones, macroalgae, speciation

## Abstract

Hybrid zones provide an ideal natural experiment to study the selective forces driving evolution of reproductive barriers and speciation. If hybrid offspring are less fit than the parental species, pre-zygotic isolating barriers can evolve and strengthen in response to selection against the hybrids (reinforcement). Four contact zones between the intertidal macroalgae *Fucus serratus* (*Fs*) and *Fucus distichus* (*Fd*), characterized by varying times of sympatry and order of species introduction provide an opportunity to investigate reinforcement. We examined patterns of hybridization and reproductive isolation between *Fs* and *Fd* in: (i) northern Norway (consisting of two natural sites, 10 000 years old), (ii) the Kattegat near Denmark (*Fd* introduced, nineteenth century) and (iii) Iceland (*Fs* introduced, nineteenth century). Using 10 microsatellites and chloroplast DNA, we showed that hybridization and introgression decreased with increasing duration of sympatry. The two younger contact zones revealed 13 and 24% hybrids and several *F*_1_ individuals, in contrast to the older contact zone with 2–3% hybrids and an absence of *F*_1_s. Cross-fertilization experiments revealed that the reduction in hybridization in the oldest zone is consistent with increased gametic incompatibility.

## 2. Introduction

The mechanisms of reproductive isolation and speciation are of central interest in evolutionary biology because of their role in determining gene flow between formerly interbreeding populations [1,2]. The last decade has seen great progress in the study of individual components of reproductive isolation and provided insights into the genetic basis of particular isolating barriers [3,4]. Yet, reproductive isolation among most species pairs is not due to a single isolating factor, but is a consequence of a large number of different pre- and post-zygotic barriers [5,6] and their potentially complex interactions. In the case of hybridizing species, reproductive barriers are more or less permeable, allowing the sexual transfer of genetic material between species (i.e. introgression). Hybrid zones, therefore, provide a natural experiment to study the selective forces causing the evolution of reproductive barriers and ultimately, the mechanisms of speciation.

Hybrid zones exist in many forms: narrow or wide, ephemeral or long lasting, andinear or mosaic [7–10]. The structure of a hybrid zone and its evolutionary fate largely depend on the fitness of the hybrid individuals relative to the parental species and three general scenarios are possible [11]. First, if there is no selection against hybridization and introgression is extensive, all individuals become hybrids, creating a hybrid swarm or ‘extinction through hybridization’ [12,13]. Second, if introgressed individuals are genetically stabilized and/or colonize new habitats, novel evolutionary lineages can arise [14–17]. Finally, if hybrid offspring are less fit than the parental species, pre-zygotic isolating barriers can evolve and strengthen in response to selection against the hybrids (i.e. increase pre-zygotic reproductive isolation) in a process called reinforcement [18].

Despite a strong theoretical foundation, the importance of reinforcement in nature remains controversial [2,19]. Reinforcement may play a larger role in speciation than previously thought [20] as it can lead to speciation of sympatric populations that have been formerly allopatric [18] or it can lead to rapid allopatric speciation [21]. Documenting reinforcement in natural populations, however, remains challenging and must address several criteria [18], including occurrence of hybridization, selection against hybrids and displacement of a heritable trait perceived by the other sex. Nevertheless, reinforcement has been documented in an increasing number of vertebrates, insects and plants [21–39]. To our knowledge, no study has examined reinforcement in other eukaryote supergroups.

The intertidal seaweed genus *Fucus* (supergroup Heterokonta; Phaeophyta) is an ideal group in which to study speciation. The genus originated in the North Pacific and after the opening of the Bering Strait (5.5–5.4 Myr BP), colonized the North Atlantic where it radiated into two distinct lineages that diverged 0.9–2.25 Myr BP: lineage 1 including *Fucus distichus*, *Fucus serratus*; and lineage 2 including *Fucus spiralis*, *Fucus vesiculosus* and others [40–45]. Each lineage is characterized by sister taxa that are dioecious with unisexual (male or female) conceptacles (e.g. *F. serratus* and *F. vesiculosus*) and hermaphrodites with co-sexual conceptacles (e.g. *F. distichus* and *F. spiralis*). The dioecy/hermaphroditism character has undergone multiple-state changes during the evolution of the genus *Fucus* [40,42,44]. Hybridization is common within the genus and molecular studies have shown that hybridization is prevalent among sister taxa (i.e. within one lineage) and mostly involves one dioecious and one hermaphrodite parental species (e.g. *F. serratus*×*F. distichus* [46,47] and *F. vesiculosus*×*F. spiralis* [48–52]). A distinguishing feature of *Fucus* hybrid zones is the limited gamete dispersal, because egg-produced pheromones effectively attract sperm only within distances of micrometres to millimetres [53] and settlement of fertilized eggs most often occurs within 1–2 m of the parent [54,55].

**Figure 1.**
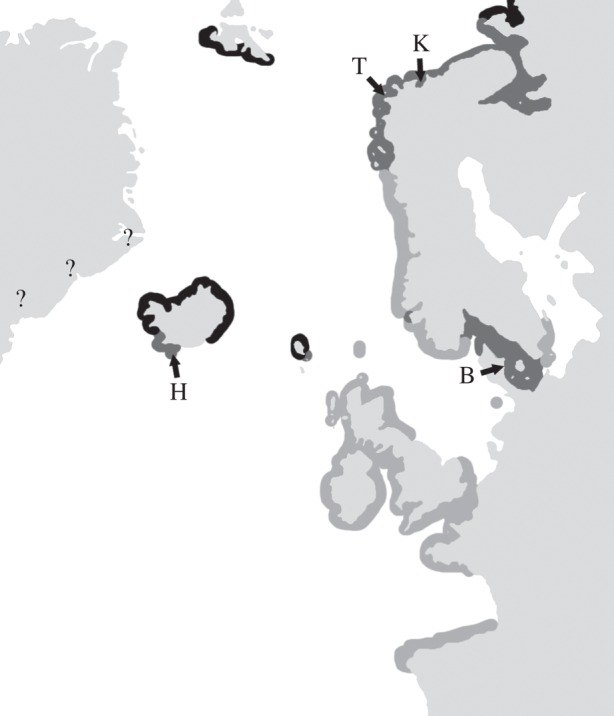
Sampling locations for *Fucus serratus* and *Fucus distichus*. Light grey depicts *F. serratus* distribution, black *F. distichus* distribution in the northeast Atlantic and dark grey sympatry. Distribution along the Greenland coast is uncertain and is designated with ‘?’.

Lineage 1 consists of the dioecious *F. serratus* (hereafter *Fs*) with a temperate east Atlantic distribution. The sister species *F. distichus* (hearafter *Fd*) is a hermaphrodite and characterized by an Arctic distribution (figure 1). The two species diverged 2.25 to 0.9 Myr ago [40,41]. Although several species have been described within the *F. distichus* lineage on the basis of morphological characteristics (e.g. [56,57]), current molecular phylogenetic evidence indicates that none form monophyletic groups and, therefore, from a phylogenetic standpoint, all are presently considered part of a single species [40,58].

Four contact zones involving *Fs* and *Fd* were identified (figure 1): (i) two in Northern Norway where the two species have been in sympatry for *ca* 10 000 years after the ice receded from the area since the Last Glacial Maximum (LGM) and *Fs* recolonized the Norwegian coast from a refugium in southwest Ireland [41]; *Fd*, on the other hand, appears to have survived the LGM in northern Norway based on mtDNA and microsatellite data [59]; (ii) Iceland (Heimaey), where *Fs* was introduced from Oslofjord (Norway), probably via imported raw wood logs, to an endemic population of *Fd* [60], leading to *ca* 100 years of sympatry; and (iii) the Kattegat Sea, where *Fd* (formerly *Fucus evanescens*) was introduced in the mid-1890s to an endemic *Fs* population [61,62], also leading to *ca* 100 years of sympatry; however, the source has not been identified owing to the lack of variability at nuclear markers (G. Hoarau and J. A. Coyer 2010, unpublished data). Crucially, the reproductive seasons of each species overlap in all zones of sympatry.

The identified contact zones between *Fs* and *Fd*, with varying times of sympatry and directionality of introduction (i.e. *Fs* to *Fd* or *Fd* to *Fs*), provide a unique opportunity to investigate the presence of reinforcement. Reinforcement is most likely to occur in areas where there is strong selection against hybrids and restricted gene flow [19], both of which are characteristic of the *Fs*/*Fd* hybrids zones [46,47]. Furthermore, as the evolution of pre-zygotic isolating barriers is expected to result in a reduction of interspecific mating through time [35], reinforcement will be stronger in older zones of sympatry. Specifically, populations in northern Norway (10 000 years of contact) should show a lower degree of hybridization and interspecific fertilization success relative to the younger hybrid zones (100 years) in the Kattegat and in Iceland.

In this study, we examined patterns of hybridization and reproductive isolation between *Fs* and *Fd* in two young and two old contact zones. Specifically, we: (i) determined the degree of hybridization and introgression using blind sampling with subsequent identification of parents and hybrids using both nuclear and chloroplast markers, and (ii) compared the degree of interspecific fertilization success within and across contact zones.

## 3. Material and methods

### 3.1 Sampling

Hybridization among *Fs* and *Fd* was examined at all contact zones: (i) Kirkenes (*n*=47) and Tromsø (northern Norway) (*n*=192), where both species are native and have been in sympatry for *ca* 10 000 years; (ii) Heimaey (Iceland) (*n*=96), where *F. serratus* was introduced *ca* 100 years ago; and (iii) Blushøj (Kattegat, Denmark) (*n*=286), where *Fd* was introduced *ca* 120 years ago. At both Tromsø and Blushøj, two 2×12 m plots were established to investigate micro-scale differences in hybridization, with the long axis perpendicular to the shoreline (beginning at the shallowest appearance of *Fd* and finishing at the deepest occurrence of *F. serratus*). Owing to physical constraints at Kirkenes and Heimaey, only one plot was established. Within each plot, a small tissue sample was excised *in situ* from all post-recruit (more than 10 cm) individuals without *a priori* species identification. Tissues were stored in silica gel crystals prior to genetic analysis. All data from Blushøj are from Coyer *et al.* [46].

### 3.2 DNA extraction and microsatellite analysis

DNA was extracted from *ca* 5 mg of silica-dried tissue as described in Hoarau *et al.* [41]. We used 10 microsatellite loci: FsA198, FsB113, FsB128, FsD39, L20, L38, L58, L94, FeF172 and FsF4 from Coyer *et al.* [46]. PCR reaction mixtures and conditions are described in Coyer *et al.* [46]. All genotypes were visualized on an ABI 377 or on a 3500XL automatic sequencer (Life technologies) and analysed with Genescan software (Life technologies).

### 3.3 Chloroplast marker

We used a portion of the ribulose-1,5-bisphosphate carboxylase operon (Rubisco; chloroplast) to identify the egg-producing parent for hybrids, as the parental species differed by a 33 bp indel [63]. PCR reactions and conditions are described in Coyer *et al.* [63] and parental identity was detected by sizing of PCR products on an ABI 377 or on a 3500XL automatic sequencer (Life technologies).

### 3.4 Molecular data analysis

Genetic diversity using Nei's [64] non-biased *H*_*exp*_, estimators of *F*_IS_ and *F*_ST_ (Wright [65]) as *f* and *θ*, [66], and allele frequencies were estimated using the software Genetix 4.02 [67]. Multilocus heterozygosity (MLH) was defined as the number of heterozygous loci per individual and ranged from 0 to 10.

Admixture of microsatellite genotypes was analysed with Structure [68], which uses a Bayesian approach to identify *K* user-defined clusters of individuals that are genetically homogeneous. Sampled individuals were assigned either to clusters or jointly to two or more clusters if their genotypes indicated admixture. All analyses were replicated 10 times for each location independently (table 1) to ensure proper convergence of the Markov chain Monte Carlo (MCMC) with the parameters: ancestry model=admixture (to account for recent divergence and shared ancestral polymorphisms); frequency model=independent; burn-in=1 000 000; MCMC length=2 000 000 post-burn-in and *K*=2 (to account for the parental species). The analysis with Structure produced for each individual, an admixture coefficient (*I*_FS_) defined as the proportion of membership to the *F. serratus* cluster.

**Table 1.** Summary of conditions and results for old and young *Fs*×*Fd* hybrid zones.

location	contact zone (time, years)	total no. samples	total % of hybrids (figure 2)	% of *F*_1_ hybrids	*F*_ST_ pure *Fd* versus pure *Fs* (all *p*<0.001)	genetic diversity (*H*_exp_)	*Fd F*_IS_ (95% CI)	interspecific fertilization success (pre-zygotic)	*F*_1_ fitness (post-zygotic)
Kirkenes NO natural	≈10 000	47	2.1	0	0.60	*Fd* 0.25 *Fs* 0.33	0.78 (0.69–0.86)	not tested	no F_1_ present
Tromsø NO natural	≈10,000	192	3.1	0	0.47	*Fd* 0.17 *Fs* 0.52	0.68 (0.58–0.76)	0–1.1%	no F_1_ present
Heimaey IC *Fs* introduced	≈100	96	23.9	8.3	0.69	*Fd* 0.10 *Fs* 0.28	0.74 (0.56–0.88)	23.7–38.3%	lower fertility survivorship not tested
Blushøj *DK*^*a*^*Fd* introduced	≈100	286	12.9	3.7	0.63	*Fd* 0.12 *Fs* 0.50	0.77 (0.69–0.84)	9.6–43.1%	lower fertility lower survivorship

^*a*^Data from [45].

The power of admixture analyses to detect hybridization in *Fucus* was evaluated by simulation using Hybridlab (v. 1.0) [69] as described previously [46]. Simulations were repeated 10 times for each location independently and these results were used to define the boundaries of the *I*_FS_ admixture coefficient (95% confidence intervals) for: (i) pure *F. serratus*, (ii) pure *F. distichus*, and (iii) *F*_1_ hybrids.

### 3.5 Fertilization success (pre-zygotic isolation)

Reproductive receptacles (consisting of 100s of conceptacles) from both species were collected within each of the contact zones (except Kirkenes). Receptacles were packed individually in aluminium foil and transported to the laboratory in a cooler box within 36–48 h. A reciprocal crossing design was used for Blushøj, Tromsø and Heimaey, using five *Fd* females, five *Fs* males, five *Fs* females and five *Fd* males, resulting in 25 *Fd* conspecific crosses, 25 *Fs* conspecific crosses and 2×25 interspecific crosses. Additionally, negative controls consisting of eggs only (no sperm added) were used for all females. All specimens were genotyped with 10 microsatellite loci and analysed as described above (e.g. Structure) to determine species identity.

Crosses were performed in sterile plastic culture plates containing 3 ml sterile seawater at 5°C and each cross and control was replicated four times using 10 oogonia (80 eggs) in each replicate. *Fs* is a dioecious species, with receptacles from a mature individual possessing conceptacles containing either antheridia or oogonia. The sex of *Fs* individuals was determined microscopically. *Fs* receptacles were stored overnight at 4°C. The addition of ice-cold sterilized seawater induced conceptacles to release eggs and sperm, which were collected and washed once in sterile seawater before further use.

As *Fd* is a hermaphroditic species with individual conceptacles within the receptacle containing both antheridia and oogonia, collecting eggs after release from conceptacles was inappropriate, because eggs from such preparations were potentially already fertilized by conspecific sperm. To prevent self-fertilization during gamete extraction from *Fd*, eggs were collected with a pipette immediately after sectioning receptacles with a razor blade in order to disrupt the conceptacles and then sequentially washed (×3) in 10 ml sterile seawater to dilute any accompanying sperm and minimize/eliminate self-fertilization. Sperm from *Fd* was also obtained by sectioning as previously described, but sections were immediately placed in a small mesh bag (50 μm mesh size) that allowed free passage of the small sperm (5–6 μm dia.), but retained the larger eggs (70–100 μm dia.). The bags were then placed in a culture dish previously inoculated with eggs for 2 h. Fertilization success was evaluated after 48 h and confirmed after one week with the development to embryos. Successes of the various combinations were compared using ANOVA and Tukey's post hoc test.

## 4. Results

### 4.1 Molecular characterization of the hybrid zones

The number of hybrids did not differ between plots within Tromsø and within Blushøj (data not shown); hence, data from both plots were subsequently pooled. Differentiation between parental species was strong with the highest *F*_ST_ estimates in the two younger contact zones (Heimaey 0.69, Blushøj 0.63) compared to the two locations in the older contact zones (Kirkenes 0.60; Tromsø 0.47) (all *p*<0.001). In all four locations, the parental species showed different levels of genetic diversity with the highest level of heterozygosity in the dioecious *Fs* and the lowest in hermaphroditic *Fd* (table 1). Heterozygote deficiencies (*F*_IS_) were equally high for *Fd* at all four locations (table 1).

**Figure 2.**
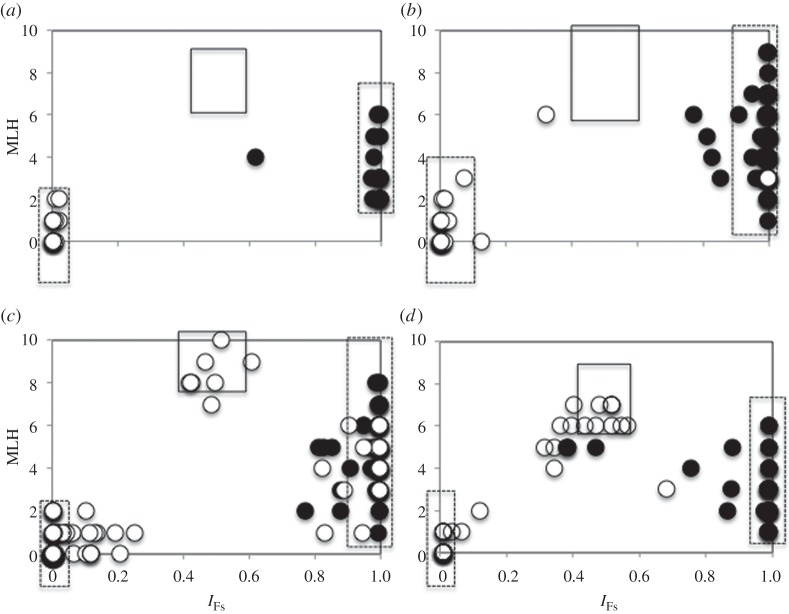
Relationship between individual MLH and introgression (*I*_FS_) for the ‘old’ ((*a*,*b*) Kirkenes and Tromsø) and the ‘young’ contact zones ((*c*,*d*) Blushøj and Heimaey). Boxes depict results from the simulation analysis: stippled=pure species; dark grey=*F*_1_ hybrids. Individuals were further classified by the origin of their chloroplasts: open circles=*F. distichus*, filled circles=*F. serratus*.

In the oldest contact zones (Kirkenes and Tromsø locations), no *F*_1_ hybrids were found and introgressed individuals accounted for only 2.1% of the 47 individuals and 3.1% of the 192 individuals, respectively (table 1 and figure 2). By contrast, 8.3% and 3.7% *F*_1_ hybrids, and 23.9% and 12.9% introgressed individuals were found in the youngest contact zones (Heimaey and Blushøj, respectively). All *F*_1_ and almost all hybrids (53 out of 68) were characterized by an *Fd* chloroplast (figure 2). In both Tromsø and Blushøj, several individuals with an *Fs* microsatellite genotype possessed an *Fd* chloroplast, but the reverse was never found.

### 4.2 Fertilization success

**Figure 3.**
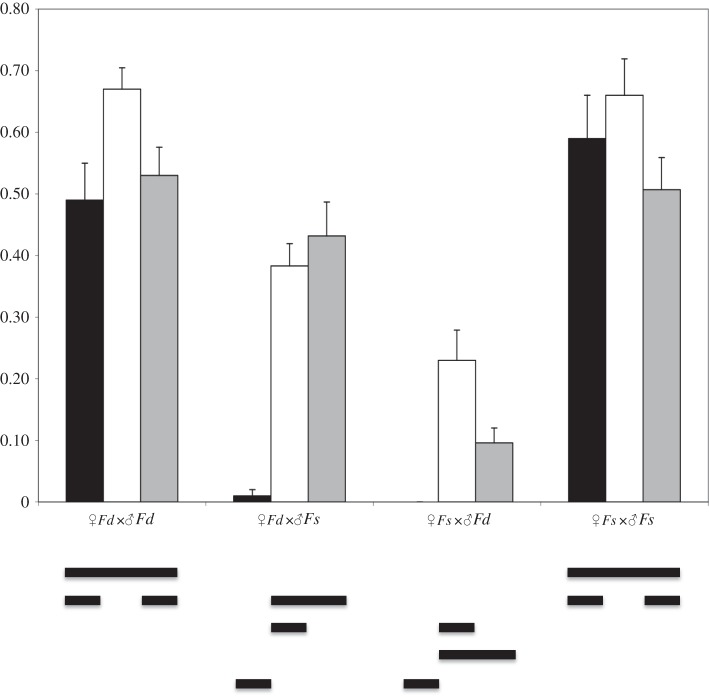
Fertilization success (the presence of one-week-old embryos) for all combinations of laboratory crosses (sample size=100). Black=Tromsø, white= Heimaey, grey=Blushøj, Kirkenes not tested. Means grouped by a horizontal line are not significantly different from each other (Tukey post hoc, *p*>0.05).

Conspecific fertilization successes (both *Fd*×*Fd* and *Fs*×*Fs*) did not differ significantly among the three contact zones examined (Kirkenes not tested) (67–49%, *p*=0.1199) (figure 3). In the young contact zones at Heimaey and Blushøj, interspecific fertilization successes involving *Fd* egg and *Fs* sperm were comparable to the conspecific success (38% and 43%, respectively), but the interspecific fertilization successes involving *Fs* egg and *Fd* sperm were significantly lower than the conspecific crosses (24% and 10%, respectively, *p*<0.001) (figure 3). In sharp contrast, both interspecific crosses showed significantly (*p*<0.001) lower success (less than 1.1%) in the oldest contact zone at Tromsø (Kirkenes not tested).

## 5. Discussion

Natural selection can drive speciation by ‘reinforcing’ those mechanisms preventing hybridization when hybrids are maladaptive. As maladaptive hybrids can be viewed as energetically expensive ‘mistakes’, pre-zygotic mechanisms may evolve to prevent hybridization. These mechanisms range from preventing mating behaviour (through enhancement of species recognition systems) to preventing gamete recognition (fertilization). In particular, pheromones and gamete surface receptor polysaccharides have been reported from a wide variety of eukaryotic species (see [33,35] and references therein) including the brown algae (fucoserratene in *F. vesiculosus* and *F. serratus*) [70]. However, these recognition molecules have primarily been studied in the context of gamete attraction rather than as possible pre-zygotic isolation mechanisms.

The pattern of greater pre-zygotic isolation in sympatry relative to allopatry is one of the main signatures of reinforcement and has been termed reproductive character displacement' [19,36]. However, establishing an empirical case for reinforcement remains difficult because isolation is not necessarily due to a single isolating factor, but a consequence of several pre- and (possibly) post-zygotic barriers and their interactions. Nevertheless, a number of criteria have been proposed to demonstrate reinforcement [18,33] including heterospecific mating (gene flow between parental species), presence of maladaptive hybrids, frequency of hybridization between populations across time, reproductive character displacement, and the absence of alternative explanations (e.g. ecological divergence via local selection or temporal isolation).

The *Fd*×*Fs* hybrid zones we examined meet some of the reinforcement criteria. First, hybridization is occurring or has occurred in all contact zones. Both younger contact zones show a high percentage of hybrids (13–24%) and several *F*_1_ individuals. Despite low contemporary hybridization in the older contact zone, there is evidence that hybridization has occurred in the past, as *ca* 3% of the individuals genotyped in the ‘old’ contact zones carry alleles from the other species (table 1 and figure 2). Furthermore, one individual with a pure *Fs* nuclear genotype and a *Fd* chloroplast DNA was found in Tromsø, resulting from past introgression.

Second, hybridization between *F*s and *Fd* appears to be maladaptive. the Blushøj *F*_1_ hybrids showed a lower fitness compared with the parental species, with selection coefficients against hybrids *ca* 80% [46] and reproductive *F*_1_ hybrids showed reduced fertility, mostly owing to lower egg quality (e.g. fewer and more variably sized eggs per receptacle). Additionally, laboratory crosses with eggs from *F*_1_ hybrids were significantly less successful both in Blushøj [47] and Heimaey where lower egg quality was also observed (J. A. Coyer and G. Hoarau 2002, unpublished data). Survivorship and fertility of *F*_1_ hybrids were not assessed at Tromsø and Kirkenes as no *F*_1_ hybrids were found in the field. In *Fucus*, as in several other species, reduction of fertility and potential sterility appear before hybrid inviability [12,71,72].

Third, patterns observed in the *Fd*×*Fs* hybrid zones are consistent with the presence of reproductive character displacement. In contrast to virtually all previous studies of reinforcement, which have compared populations of plants and animals in regions of sympatry to those in allopatry, we examined sympatric populations differing in the time of sympatry (100 to 10 000 years), as reinforcement is expected to result in a reduction of interspecific mating through time ([35] and references therein). Our molecular data suggest that hybridization has decreased with the time of contact. Although based on only two locations per age, both of the younger contact zones revealed a high percentage of hybrids (13–24%) and the presence of *F*_1_ individuals. By contrast, hybridization was very low (2–3%) and no *F*_1_ individuals were found in either of the old contact zones in northern Norway (table 1 and figure 2). Our cross-fertilization experiments further suggested that the reduction in hybridization observed in Tromsø was the result of increased gametic incompatibilities. Whereas interspecific fertilization success (*Fd* egg and *Fs* sperm) was comparable to conspecific crosses in the younger contact zones, interspecific fertilization successes decreased to virtually zero in Tromsø (figure 3). While our results cannot provide conclusive evidence for reinforcement (i.e. increased isolation in sympatry compared with allopatry using the temporal comparison) because of low replicates, they are consistent with patterns that would be expected over such a period of time.

In this context, we reviewed a new contact zone in Bergen Harbour, where *Fd* was introduced to *Fs* within the past 10 years, where we expected to see many hybrids. Unexpectedly, a genetic survey conducted in 2008 found no hybrids (G. Hoarau and J. A. Coyer 2008, unpublished data). At least three aspects of the Bergen population, however, can temper the contradiction. First, the source of *Fd* to Bergen Harbour is unknown and to date, impossible to trace (G. Hoarau and J. A. Coyer 2008, unpublished data). The source of *Fd* in Bergen could be northern Norway and subsequently, a population already displaying strong reinforcement. Second, the phenological overlap in Bergen Harbour is reduced (K. Sjøtun 2008, personal communication) compared with all other zones of contact, thus limiting the temporal window for hybridization. This difference in observed phenology at Bergen, in fact, suggests a northern origin of *Fd* [73]. Finally, *F. distichus* is an order of magnitude more abundant than *F. serratus* in Bergen Harbour (J. A. Coyer, K. Sjøtun and G. Hoarau 2008, unpublished data), thus further limiting the likelihood of hybridization (which requires threshold densities of gametes). These results further highlight the difficulties in definitively proving reinforcement. Nevertheless, we consider the temporal approach involving contact zones compelling (especially, the older ones).

Ecological divergence caused by strong local selection is also a potentially contributing mechanism, especially when gamete recognition systems are weak (reviewed in [74], see also [75,76]). Although *Fd* and *Fs* can differ in position on the intertidal shore, with *Fd* occurring slightly higher on the shore than *Fs*, wide areas of intermixing were the rule at all four contact zones and our sampling occurred within the areas of intermixing (table 1). Furthermore, no pattern of microhabitat utilization was detected in a detailed spatial analysis of the parental species and *F*_1_ hybrids at Blushøj: mature plants were intermingled and *F*_1_ hybrids occurred throughout the intermingled area rather than clustered around either parental species or themselves [46]. Similar observations were made at Tromsø (J. A. Coyer and G. Hoarau 2010, unpublished data). Nevertheless, microhabitat differentiation cannot be entirely ruled out. In addition, no phenological differences were apparent as reproductive seasons of both parental species overlapped in all zones of sympatry and sexually mature individuals of both species were abundant when we sampled (G. Hoarau and J. A. Coyer 2010, personal observation); contrary to the situation in Bergen.

Changes in the mating system, such as increased/decreased self-fertilization in plants, can be another mechanism for reproductive character displacement (reviewed in [36]). Selfing may thus promote pre-zygotic isolation. In the hermaphroditic *Fd*, high inbreeding coefficients (up to 10× higher than *Fs*) have been routinely found in broad surveys (Coyer *et al.* [59]), whereas in the dioecious *Fs*, this is seldom the case (Coyer *et al.* [47]). However, no evidence for mating system change was observed, as inbreeding coefficients (*F*_IS_) for *Fd* were not significantly different among the contact zones (table 1) and increased levels of self-fertilization did not occur. As shown in this study, as well as Coyer *et al.* [46], hybridization is asymmetrical with *Fd* providing the egg most of the time (figure 3). To our knowledge, no model of hybrid zones has considered a mixed mating system and little more can be said at the present time. What we do know, however, is that hybrids are almost never formed between *Fucus* species with the same mating systems.

We hypothesized that sexual trait(s) accounting for reinforcement in *Fucus* would most likely occur at the level of gamete attraction and/or recognition. Gamete recognition proteins/genes in marine organisms have been investigated mainly in marine invertebrates (mussels, snails and sea urchins) [76,77]. In these systems, positive selection (as assessed by high sequence divergence and more non-synonymous than synonymous substitutions) for sperm-egg interaction proteins has been found in closely related and sympatric species of sea urchins [78,79], but not for species in allopatry [80]. Sympatry among closely related species also seems to be associated with increased sperm specificity. In sea urchins, for example, crosses between allopatric species pairs require substantially less sperm for fertilization than crosses involving sympatric pairs [81]. The sperm binding protein *fss27* in *Fucus* has long been known and shares a number of chemical properties with sea urchin bindin gamete recognition protein [82–84]. Recognition between eggs and sperm has been hypothesized to be based upon a receptor mechanism involving ligands (oligosaccharide side-chains of egg surface glycoproteins) and complementary binding proteins [84]. However, the protein and its ligand remain to be sequenced.

Another important evolutionary mechanism potentially occurring at the level of gamete attraction/recognition is sexual conflict [85,86]. For example, an overabundance of sperm increases the risk of polyspermy (multiple sperm entry), which will prevent subsequent development of embryos. Under these conditions, male and female gametes have different priorities: males for fast entry into eggs, females for prevention of polyspermy and slow sperm entry. Any mutation that makes it more difficult to fertilize eggs can concomitantly lower the effective concentration of sperm, thereby allowing eggs to block polyspermy. Thus, a conflict over fertilization rates arises in which females are selected for lower egg–sperm affinity, whereas males are selected for higher affinity. This affinity ‘arms race’ could lead to divergences in the gamete recognition system and thus to reproductive character displacement, independent from reinforcement [76,87].

*Fucus* species display a fast and sodium-dependent block to polyspermy [88,89]. Consequently, polyspermy rates in natural populations are higher in brackish (*F. vesiculosus* [90]) relative to fully marine habitats (*F. ceranoides* [91], *F. distichus* [92]). Three of the contact zones examined in this study (Tromsø, Kirkenes and Heimaey) were fully marine and although the Blushøj site could vary between near fully marine (30 psu) to semi-brackish (20 psu) over a time scale of hours to days, polyspermy in *F. vesiculosus* most commonly occurs at less than 6.5 psu [90]. Consequently, polyspermy is unlikely to drive sexual conflict among *Fd* and *Fs* gametes in the contact zones we examined. An additional outcome of divergence from sexual conflict is lower conspecific fertilization success (reviewed in [36]), which was not observed in our study. Therefore, the most parsimonious explanation for the pattern we observed in *Fd*×*Fs* hybrid zones is probable reinforcement of pre-zygotic isolation mechanisms.

## 6. Conclusion

The genus *Fucus*, with the combination of contemporary radiation (e.g. *F. radicans* within the past 400–2000 years; [93], secondary contact zones (e.g. Coyer [46]), extensive hybridization (e.g. [46,50]), and a well-supported phylogenetic [40,42,44] and phylogeographic framework [18,19], e.g. *F. serratus* [41], e.g. *F. distichus* [58], e.g. *F. vesiculosus* and *F. spiralis*[43]) provides evolutionary biologists with a system phylogenetically distant from, but complementary to, plants and animals. While the results of our study of *Fd*×*Fs* hybrid zones do not prove reinforcement, they are consistent with reinforcement of pre-zygotic isolation as shown by the decreasing rates of hybridization and interspecific fertilization success with increasing time of sympatry. Evidence for reinforcement will be most compelling when observed in species pairs with a well-corroborated historical biogeographical framework, as it the case in *Fucus* (e.g. [21]).

## Supplementary Material

Genotypes for the Fucus contact zones

## Data accessibility

The supporting data can be found in the electronic supplementary material.
